# Risk and prognostic factors for SARS-CoV-2 infection in Spanish population with multiple sclerosis during the first five waves

**DOI:** 10.3389/fneur.2022.1001429

**Published:** 2022-10-19

**Authors:** Belén Pilo De La Fuente, Julio González Martín-Moro, Guillermo Martín Ávila, Alejandro Méndez Burgos, Laura Ramos Barrau, Israel Thuissard, Marta Torrejón Martín, Yolanda Aladro Benito

**Affiliations:** ^1^Department of Neurology, Getafe University Hospital, Madrid, Spain; ^2^Faculty of Biomedical and Health Sciences, European University of Madrid, Madrid, Spain; ^3^Department of Ophthalmology, Hospital Universitario del Henares, Madrid, Spain; ^4^Faculty of Medicine, Universidad Francisco de Vitoria, Madrid, Spain

**Keywords:** incidence, multiple sclerosis, COVID-19, severity, coronavirus

## Abstract

**Background:**

Data on coronavirus disease 2019 (COVID-19) incidence in patients with multiple sclerosis (MS) during the first wave have been published but are scarce for the remaining waves. Factors associated with COVID-19 infection of any grade are also poorly known. The aim of this study was to analyze the incidence, clinical features, and risk factors for COVID-19 infection of any grade in patients with MS (pwMS) during waves 1–5.

**Methods:**

This study prospectively analyzes the cumulative incidence of COVID-19 from the first to the fifth waves by periodic case ascertainment in pwMS followed at the University Hospital of Getafe (UHG). Global and stratified cumulative incidence was calculated. Logistic regression models were used to estimate the weight of selected variables as risk and prognostic factors.

**Results:**

We included 431 pwMS, of whom 86 (20%) were infected with severe acute respiratory syndrome coronavirus 2 (SARS-CoV-2). The overall cumulative incidence of confirmed cases was similar to that of Madrid (13,689 vs. 13,307 per 100,000 habitants) but 3 times higher during the first wave and slightly lower from the second to the fifth waves. The majority (86%) of pwMS developed mild forms of COVID-19. Smoking was the only factor associated with a decreased risk of SARS-CoV2 infection of any grade [odds ratio (OR) 0.491; 95% CI 0.275–0.878; *p* = 0.017]. Risk factors associated with severe forms were Expanded Disability Severity Scale (EDSS) ≥3.5 (OR 7.569; 95% CI 1.234–46.440) and pulmonary disease (OR 10.763; 95% CI 1.27–91.254).

**Conclusion:**

The incidence of COVID-19 was similar in this MS cohort to the general population. Smoking halved the risk of being infected. Higher EDSS and pulmonary comorbidity were associated with an increased risk of severe forms.

## Introduction

In December 2019, severe acute respiratory syndrome coronavirus 2 (SARS-CoV-2) was first reported in Wuhan, China (1). Since then, it has spread rapidly around the world causing significant morbidity and mortality. Patients with multiple sclerosis (pwMS) represent a population of particular interest in this pandemic context due to the nature of their disease and the use of a wide range of immunologically active drugs (2). On the one hand, disease-modifying therapies (DMTs) have immunosuppressive effects that could hamper an effective immune response to the infection (3, 4). On the other hand, immunosuppression could offer protection by downregulating hyperinflammation and the cytokine storm associated with coronavirus disease 2019 (COVID-19) (5–7).

The Multiple Sclerosis International Federation has made recommendations regarding the risk of COVID-19 in pwMS, with a specific statement on DMTs (Multiple Sclerosis International Federation, 2021, https://www.msif.org). In addition, national MS societies have published guidelines that stratify the risk of the accepted treatments (8, 9).

Very few population-based studies on the incidence of COVID-19 in pwMS compared to the general populations have been published (10). Two recent studies, from Scotland and Brazil, have reported similar incidences of infection in pwMS and the general population (11, 12).

Older age, male sex, comorbidities (e.g., obesity, diabetes, hypertension, cardiovascular, and pulmonary disease), non-ambulatory status, and progressive forms have been suggested as risk factors for severe forms of COVID-19 (3, 13, 14). However, risk factors for developing any degree of SARS-CoV-2 infection in pwMS are unknown. Therefore, assessing the risk of COVID-19 in these patients is an important public health issue (15).

The purpose of this study was to compare the cumulative incidence of COVID-19 in pwMS and in the general population of Madrid from the first to fifth waves and compare it to the cumulative incidence in the general population of Madrid region and determine risk and prognostic factors associated with infection in these patients.

## Methods

### Study design and population

We conducted an ambispective (retrospective during the first wave, and prospective from second to fifth waves) observational study on all pwMS and other demyelinating disorders currently followed at the University Hospital of Getafe (UHG). UHG is a Public Health Hospital located in the south of the community of Madrid with an assigned population of 226,666 habitants (https://www.comunidad.madrid/hospital/getafe). It has a multiple sclerosis (MS) expertise unit since more than 20 years ago.

All patients with the following diagnosis were included: radiologically isolated syndrome (RIS), clinically isolated syndrome (CIS), relapsing-remitting MS (RRMS), secondary progressive MS (SPMS), or primary progressive MS (PPMS).

The sources of COVID-19 ascertainment were (a) asking patients during their scheduled MS consultation, usually every 6 months; and (b) periodic review of all medical documentation (neurological, emergency, and primary treating physician's reports) existing in the Public Health System since 1 March 2020. All Public Hospitals in Madrid Community are connected to each other and to the primary treating physicians through a well-developed computerized network. In this network, all emergency care reports, as well as all testing for SARS-CoV2 performed in the Public Health System, and primary treating physician's reports are available. A final review of medical reports of all patients was performed at the close of the study on 28 September 2021.

The study adhered to the Declaration of Helsinki principles was approved by the Getafe University Hospital Ethics committee and did not receive any financial support.

### Selected variables

We collected patients' baseline characteristics on 1 March 2020: Expanded Disability Severity Scale (EDSS) score at baseline, number of relapses in the previous year, radiological activity (defined as the presence of new T2 lesions and/or T1 gadolinium-enhanced lesions), current DMT use, current lymphocyte count, and comorbidities (hypertension, diabetes, dyslipidemia, smoking, cardiovascular, and pulmonary disease).

Lymphopenia was defined as grade 1: absolute lymphocytes count (ALC) of 800–999/μl; grade 2: ALC of 500–799/μl; grade 3: ALC of 200–499/μl; and grade 4: ALC of < 200/μl.

Disease-modifying therapies were grouped according to potential infection risk (no risk: interferon beta and glatiramer acetate; low risk: teriflunomide, azathioprine, dimethyl fumarate, and natalizumab; intermediate or high risk: fingolimod, anti-CD20 therapies, cladribine, and alemtuzumab), as proposed in previous studies (14, 16).

Following the European Center for Disease Prevention and Control Guidance, patients with fever, dyspnea, cough, or sudden onset of anosmia, ageusia, or dysgeusia after February 2020 were considered possible cases; those with clinical criteria, radiological criteria (ground-glass opacities), or an epidemiological link were defined as probable cases and those with a positive SARS-CoV-2 laboratory test [polymerase chain reaction (PCR) or rapid nucleocapsid protein antigen detection (RAD)] in nasopharyngeal swab or with demonstrated SARS-CoV-2 antibodies (IgG or IgM) in a blood sample) were established as confirmed cases (*Case Definition of Coronavirus 2019 (COVID-19) as of 3 December 2020*. https://www.ecdc.europa.eu/En/Covid-19/Surveillance/Case-Definition. *Accessed 27 November 2021*., n.d.). Patients were reclassified according to the COVID-19 case definition in confirmed or suspected (which included possible and probable) cases.

Regarding COVID-19, we collected symptoms and laboratory and radiological results. To study the severity of infection, we used the COVID-19 severity score proposed by Louapre et al. (14) based on a 7-point ordinal scale in which 1 indicated that the patient was not hospitalized and had no limitations on activities; 2 indicated that the patient was not hospitalized but had a limitation on activities; 3 indicated that the patient was hospitalized but did not require supplemental oxygen; 4 indicated that the patient was hospitalized and required supplemental oxygen; 5 indicated that the patient was hospitalized and received non-invasive ventilation or high-flow oxygen; 6 indicated that the patient was hospitalized and received invasive mechanical ventilation or extracorporeal membrane oxygenation; and 7 indicated death. The mild disease was defined as patients who did not require hospitalization (Louapre severity scores of 1 and 2), and moderate-severe disease was considered when patients were hospitalized (Louapre severity scores of 3–7).

The date of diagnosis was also collected and the first five waves in Spain were included until 28 September 2021 (first wave from 1 March 2020 to 30 June 2020; second wave from 1 July 2020 to 1 December 2020; third wave from 2 December 2020 to 9 March 2021; fourth wave from 10 March 2021 to 22nd June 2021; fifth wave from 23 June 2021 to 28 September 2021).

In addition, the SARS-CoV-2 vaccination history was reviewed since the COVID-19 vaccination campaign in Spain began on 1 January 2021 (vaccine brand and date of vaccination if applicable were recorded).

### Statistical analysis

Baseline data were compared between patients who were infected with SARS-CoV-2 (COVID+) and those who were not (COVID–). Group comparisons were performed using chi-square (χ^2^) (or Fisher's exact test) for categorical data and Student's *t*-test (or Mann–Whitney *U* test) for continuous data. Any two-sided *p* < 0.05 was considered statistically significant.

Univariate and multivariate logistic regression models were developed to assess the association between demographic and clinical characteristics with SARS-CoV-2 infection and with the severity of COVID-19 (mild vs. moderate-severe). Age, sex, MS phenotype (relapsing vs. progressive), EDSS, clinical and radiological activity, DMT level, and comorbidities were entered into the model. Variables with *p*-values ≤ 0.1 in the univariate analysis were entered into the multivariate model and those with *p*-values ≤ 0.05 were retained. Results were expressed as odds ratios (OR) and 95% CIs.

A Cox model was developed to assess the association between demographic and clinical characteristics with SARS-CoV-2 infection.

Data were analyzed using the Statistical Package for Social Sciences, version 22.0 (IBM SPSS, Inc., Chicago, IL, USA).

Given that Getafe belongs to the community of Madrid, we used the Madrid population as a reference. Cumulative incidence in Madrid was extracted from the official epidemiologic database from the city generated by a local health system, which is updated weekly (https://www.comunidad.madrid/covid-19). As at the beginning of the pandemic diagnostic capacity was very limited, first-wave data were less valuable. For this reason, the Madrid health council analyzed separately the first and posterior waves. Global cumulative incidence until 28 September 2021 has been calculated in the MS cohort and in the general Madrid population only with confirmed cases (suspected cases were not registered in Madrid).

For second and posterior waves, cumulative incidence was adjusted by sex and age to the Madrid population. Data series of Madrid confirmed cases (positive PCR, antigen test, or antibody test) were analyzed independently and compared with data obtained from the MS cohort.

Epidemiological analysis of the data was done using Excel (Microsoft 365 MSO version 2201).

## Results

On 28 September 2021, 431 pwMS-related disorders were followed at UHG. The baseline characteristics of the cohort are summarized in Table 1 (in global population, as well as in COVID+ and COVID–). Briefly, 299 (69.4%) of pwMS were women. The median age was 47.1 years (range 18–81.9), median disease duration was 12.3 years (range 0–47.9), and median EDSS score was 2 (range 0–9.5). A total of 85 (19.7%) received intermediate or high-risk DMT,88 (20.4%) had progressive forms of the disease, and 264 (61.3%) had no evidence of disease activity (NEDA-3). Comorbidities and basal lymphocyte count of the cohort are detailed in Table 1. A lower percentage of smoking patients was found in COVID+ population (21.3 vs. 35.5% in patients with COVID, *p* = 0.017). No other significant differences were found.

**Table 1:** Demographic, clinical characteristics, comorbidities, and basal lymphocyte count of pwMS-related disorders followed in University Getafe Hospital, as of 28 September 2021.

**Characteristics**	**Global population** **(*n* = 431)**	**COVID+ population** **(*n* = 86)**	**COVID- population** **(*n* = 345)**	***p*-value**
Female, *n* (%)	299 (69.4)	62 (72.1)	237 (68.7)	*0.602*
Age at pandemic onset, median (range)	47.1 (19–81.9)	46 (19–80.1)	47.6 (19–81.9)	*0.485*
Disease duration, y, median (range)	12.3 (0–47.9)	12.4 (0–47.9)	12.2 (0–47.2)	*0.501*
Last EDSS score, median (range)	2 (0–9.5)	2 (0–9.5)	2 (0–9.5)	*0.719*
Disease-modifying treatment, *n* (%)				*0.945*
-No treatment	138 (32)	30 (34.9)	108 (31.3)	
-No risk treatment	98 (22.7)	19 (22.1)	79 (22.9)	
-Low-risk treatment	110 (25.5)	21 (24.4)	89 (25.8)	
-Moderate-high risk treatment	85 (19.7)	16 (18.6)	69 (20)	
MS type, *n* (%)				*0.631*
-RRMS	321 (74.5)	69 (80.2)	252 (73)	
-SPMS	66 (15.3)	12 (14)	54 (15.7)	
-PPMS	22 (5.1)	3 (3.5)	19 (5.5)	
-CIS	14 (3.2)	2 (2.3)	12 (3.5)	
-RIS	8 (1.9)	0 (0)	8 (2.3)	
Disease activity, *n* (%)				
-patients with MS relapses	51 (11.8)	9 (10.4)	42 (12.2)	*0.852*
-patients with EDSS progression	73 (16.9)	16 (18.4)	(16.5)	*0.632*
-patients with radiological activity	109/413 (27)	20/82 (24.4)	89/331 (26.9)	*0.678*
-patients with NEDA-3	264 (61.3)	47 (54.7)	217 (62.9)	*0.174*
Comorbidities, *n* (%)				
-Smoking	134/410 (31.1)	17/80 (21.3)	117/330 (35.5)	*0.017*
-Hypertension	71 (16.5)	11 (12.8)	60 (17.4)	*0.335*
-Diabetes	18 (4.2)	2 (2.3)	16 (4.6)	*0.546*
-Cardiovascular disease	11 (2.6)	3 (3.5)	8 (2.3)	*0.465*
-Pulmonary disease	30 (7)	7 (8.1)	23 (6.7)	*0.637*
Basal lymphocyte count, *n* (%)				*0.163*
-No lymphopenia	364/429 (84.8)	69 (80.2)	295/343 (86)	
-Lymphopenia grade 1	30/429 (7)	7 (8.1)	23/343 (6.7)	
-Lymphopenia grade 2	24/429 (5.6)	6 (7)	18/343 (5.2)	
-Lymphopenia grade 3	11/429 (2.6)	4 (4.7)	7/343 (2)	
-Lymphopenia grade 4	0 (0)	0 (0)	0 (0)	

EDSS, Expanded Disability Status Scale; MS, multiple sclerosis; RRMS, relapsing-remitting multiple sclerosis; SPMS, secondary progressive multiple sclerosis; PPMS, primary progressive multiple sclerosis; CIS, clinically isolated syndrome; RIS, radiologically isolated syndrome; NEDA-3, no evidence of disease-activity-3.

### Characteristics of SARS-CoV-2 infection in the MS cohort

**Figure 1:**
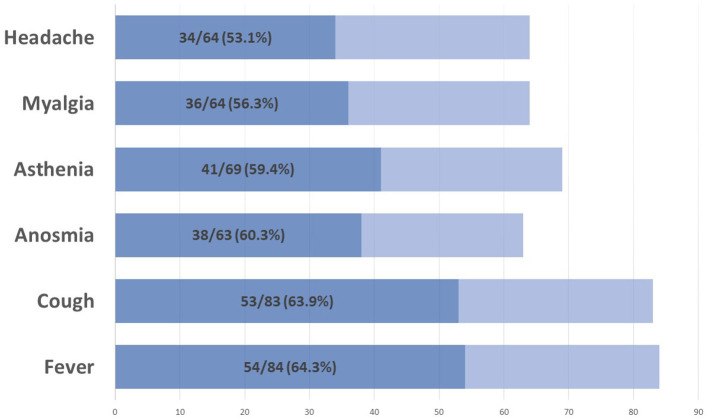
Main COVID-19 symptoms.

**Figure 2:**
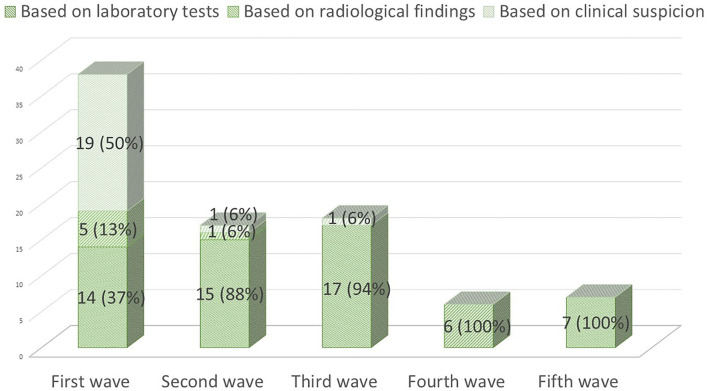
COVID-19 cases during the first five waves (1 March 2020 to 28 September 2021) and diagnostic criteria used.

**Figure 3:**
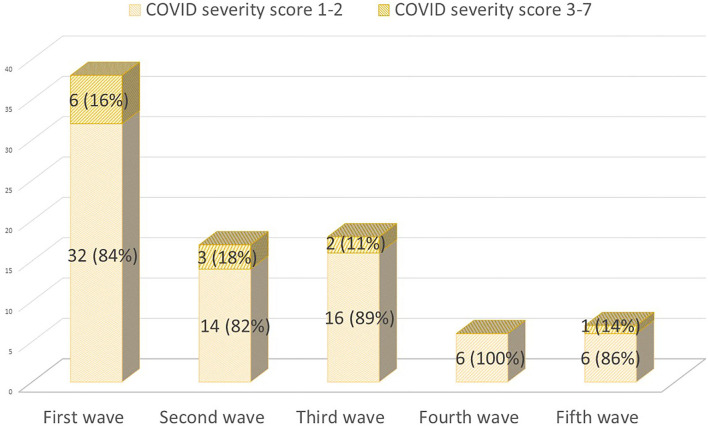
COVID-19 cases during the first five waves (1 March 2020 to 28 September 2021) classified by their severity [(14); severity scale: 1–2 mild cases; 3–7 severe cases who required hospitalization].

On 28 September 2021, 86 (20%) pwMS had suffered from COVID-19 since the beginning of the pandemic. Figure 1 summarizes the main symptoms, being fever (64.3%) and cough (63.9%) the most frequent ones. Of these 86 patients, 59 (68.6%) were confirmed cases and 27 (31.4%) were suspected COVID-19 cases. Figure 2 represents the number of cases of each wave, with cases broken down by the referred diagnostic criteria. Most suspected cases 24/27 (89%) belong to the first wave due to the limited availability of tests during this period. Only 12 (14%) of all cases suffered severe forms that required hospitalization, and half of them were infected during the first wave. Only one of the 431 patients died (during the second wave). None of the patients needed intensive care unit admission. Only one patient (1.2%), who was infected during the second wave (1.2%), required high-flow oxygen. Figure 3 represents the evolution of severity along different waves.

One (1.2%) patient had COVID-19 twice (during first and second waves). Between January 2021 (when the vaccination campaign started in Madrid) and 28 September 2021, 88.4% (375/424) of pwMS were completely vaccinated. Four (4.7%) patients had COVID-19 after vaccination, and one of them had 2 weeks after the first dose. None of them received moderate or high-risk treatment (one had no treatment, two had interferons, and one had glatiramer acetate).

### Cumulative incidence of SARS-CoV-2 infection

The cumulative incidence of confirmed cases in the MS cohort until 28 September 2021 (including five waves) was similar to that of the Madrid general population (13,689 per 100,000 habitants vs. 13,307 per 100,000 habitants, *p* = 0.815) (Table 2A).

**Table 2A:** Sex-adjusted cumulative incidences of COVID-19 in our MS cohort and in Madrid population during the first wave, during second to fifth waves, and global (first to fifth waves).

**Age group**	**Sex**	**Cumulative incidence in Madrid (1st wave) per 100,000 hab.** **(95% IC)**	**Cumulative incidence in MS (1st wave) per 100,000 hab.** **(95% IC)**	**Cumulative incidence in Madrid (2nd−5th waves) per 100,000 hab.** **(95% IC)**	**Cumulative incidence in MS (2nd−5th waves) per 100,000 hab.** **(95% IC)**	**Global cumulative incidence in Madrid per 100,000 hab.** **(95% IC)**	**Global cumulative incidence in MS group per 100,000 hab.** **(95% IC)**
Total	M	985 (974–996)	2,273 (469–6,642)	12,373 (12,335–12,411)	12,121 (6,928–19,684)	13,358 (13,319–13,398)	14,394 (8,666–22,478)
	F	1,068 (1,057–1,079)	3,679 (1,837–6,583)	12,191 (12,155–12,228)	9,699 (6,496–13,929)	13,260 (13,323–13,298)	13,378 (9,557–18,217)
	Total	1,029 (1,021–1,036)	3,248 (1,776–5,450)	12,278 (12,252–12,305)	10,441 (7,616–13,971)	13,307 (13,279–13.334)	13,689 (10,421–17,658)

During the first wave, the cumulative incidence was three times higher in the population with MS (3,248 vs. 1,029, *p* < 0.001; men 2,273 vs. 985; women 3,679 vs. 1,068) (Table 2A). During the second and posterior waves, the cumulative incidence was slightly lower in the MS cohort than in the general population but not statistically significant (10,441 vs. 12,278, *p* = 0.245) (Table 2A).

In the analysis of age and sex-adjusted cumulative incidences of confirmed cases during the second to fifth waves, significant heterogenicity was found among different age groups (Table 2B). For instance, the higher cumulative incidence was found in men aged between 25 and 44 years in the MS group (18,750 vs. 14,247 in the general population), whereas the lower cumulative incidence was observed in women aged above 65 years in the MS group (3,333 vs. 7,937 in the general population).

**Table 2B:** Age and sex-adjusted cumulative incidences of COVID-19 in our MS cohort and in Madrid population during second to fifth waves.

**Age group**	**Sex**	**Cumulative incidence in Madrid** **(2nd−5th waves) per 100,000 habitants (95% IC)**	**Cumulative incidence in MS group** **(2nd−5th waves) per 10,000 habitants (95% IC)**
15–24	M	18,604 (18,461–18,749)	–
	F	19,462 (19,314–19,611)	–
	Total	19,029 (18,925–19,132)	–
25–44	M	14,247 (14,172–14,323)	18,750 (8,574–35,593)
	F	14,582 (14,507–14,658)	13,008 (7,435–21,124)
	Total	14,418 (14,365–14,472)	14,620 (9,461–21,582)
45–64	M	11,401 (11,332–11,470)	8,824 (3,238–19,205)
	F	11,316 (11,250–11,381)	8,633 (4,461–15,080)
	Total	11,357 (11,309–11,404)	8,696 (5,154–13,743)
≥65	M	8,590 (8,509–8,671)	7,692 (195–42,859)
	F	7,937 (7,871–8,003)	3,333 (84–18,572)
	Total	8,208 (8,157–8,259)	4,651 (563–16,802)
Total	M	12,373 (12,335–12,411)	12,121 (6,928–19,684)
	F	12,191 (12,155–12,228)	9,699 (6,496–13,929)
	Total	12,278 (12,252–12,305)	10,441 (7,616–13,971)

### Variables associated with COVID-19 infection

**Figure 4:**
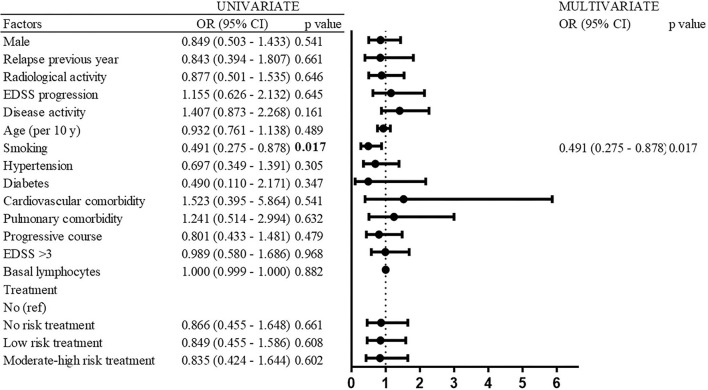
Risk factors of SARS-CoV-2 infection (univariate and multivariate analyses).

Only smoking was associated with a decreased risk of SARS-CoV-2 infection (OR 0.491; 95% CI 0.275–0.878; *p* = 0.017). No other factors, neither demographic, clinical, disability, comorbidity, nor treatment was associated with the risk of SARS-CoV-2 infection (Figure 4).

**Figure 5:**
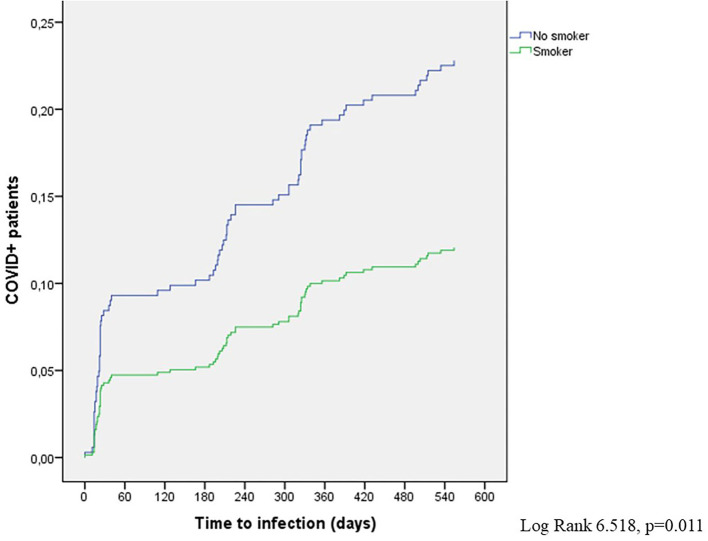
COVID+ patients in the smoker and non-smoker subgroups (Cox model).

The Cox proportional hazard regression analysis replicated the results of the logistic regression model. Again, only smoking was associated with a reduced risk of SARS-CoV-2 infection [hazard ratio (HR) 0.497; 95% CI 0.287–0.866; *p* = 0.012] with 77.2% patients with COVID in the non-smoker group and 88% patients with COVID in the smoker group as of 28 September 2021 (Figure 5).

### Variables associated with severe COVID-19

**Figure 6:**
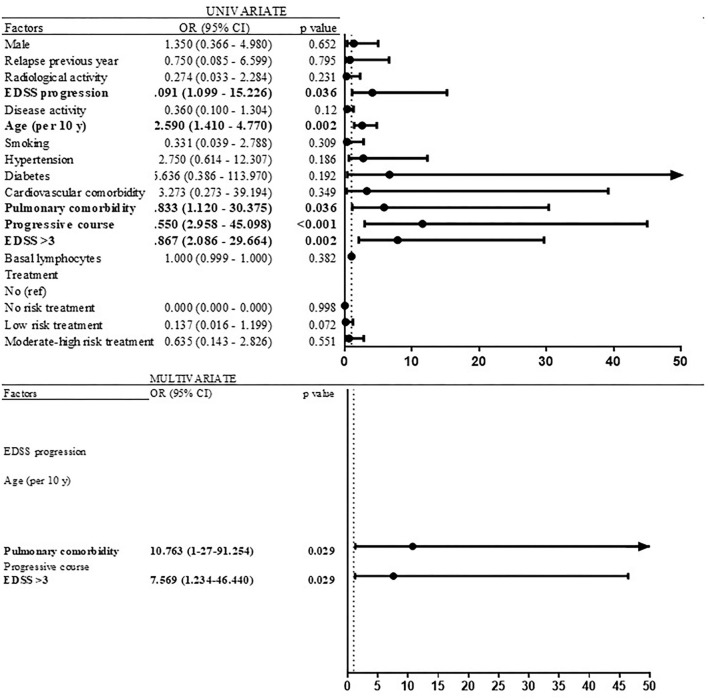
Risk factors of severe COVID-19 (univariate and multivariate analyses).

Significant risk factors associated with severe forms of COVID-19 (that required hospitalization) in the univariate logistic regression models were EDSS progression (OR 4.091; 95% CI 1.099–15.226; *p* = 0.036), age (OR per 10 years 2.594; 95% CI 1.411–4.766; *p* = 0.002), pulmonary comorbidity (OR 5.833; 95% CI 1.120–30.375; *p* = 0.036), progressive course (OR 11.55; 95% CI 2.958–45.098; *p* < 0.001), and EDSS ≥3.5 (OR 7.867; 95% CI 2.086–29.664; *p* = 0.002) (Figure 6). In the multivariate logistic regression model, EDSS ≥ 3.5 (OR 7.569; 95% CI 1.234–46.440; *p* = 0.029), pulmonary comorbidity (OR 10.763; 95% CI 1.27–91.254; *p* = 0.029), and age (OR per 10 years 1.692; 95% CI 0.886–3.219; *p* = 0.113) were retained (Figure 6).

## Discussion

This observational study of COVID-19 incidence in a cohort including all pwMS followed in a hospital of Madrid has many strengths. First, case ascertainment bias is likely to be small because it is based on a systematic review of all patients in the cohort. They were regularly interrogated about their COVID-19 experience during their medical visits and, aside from patients' anamnesis, primary physician's medical, emergency, and hospitalization reports were reviewed periodically until 28 September 2021, to detect unreported mild cases and to integrate all cases up to that date. Second, this analysis has incorporated data until 28 September 2021, including the first five waves, which enables us to analyze the pandemic evolution in our MS cohort with more accurate data due to the higher percentage of confirmed cases and its chronological comparison with the vaccination campaign in Madrid.

We found a cumulative incidence of confirmed cases in the MS cohort until 28 September 2021, similar to that of the Madrid population, higher during the first wave and lower in the second and subsequent waves. Sepúlveda et al. also found an incidence of confirmed COVID-19 similar to that of the Barcelona population (16) and a 2-fold higher cumulative incidence when all cases (confirmed and suspected) were included. Nevertheless, their data are restricted to the first wave until 18 June 2020. In this period, we found a 3-fold higher cumulative incidence than that observed in the Madrid population, including only confirmed cases. These differences may be explained by the different case ascertainments, based on questionnaires completed by patients vs. systematic and periodic reviews of medical records and direct interviews with patients in our study.

Crescenzo et al. reported a frequency measure of confirmed COVID-19 in pwMS 2.5 times higher than that reported in the inhabitants of the Veneto region (1 vs. 0.4%) (17). Infection rates during the first wave are not comparable for multiple reasons, and the pressure of care and availability of diagnostic tests differ in each country and regions of each country. The higher incidence of confirmed infection in MS cohorts could be explained in part by increased surveillance and testing to optimize the surveillance of patients, theoretically more susceptible to severe infection.

In the analysis of the waves' evolution since the beginning of the pandemic, we can observe a progressive reduction in the proportion of suspected cases and a progressive increase in the percentage of confirmed cases that can account for the lack of diagnostic tests during the first months and its posterior progressive availability. During the first wave, only 37% of our patients had a positive confirmatory laboratory test, data slightly inferior to the percentages observed in other series: 45.3% (39 out of 86 patients) in the Dutch cohort (13) and 42.1% (146 out of 347) in the French registry (14). The different methodology used (register vs. systematic review of a cohort) and the scarce availability of tests during that period (in which laboratory tests were usually reserved for more severe cases) may account for the differences observed.

In addition, the analyses of the waves' evolution show a progressive downward trend in the number of infected patients, more pronounced from the third wave onward, coinciding with the beginning of the vaccination campaign in Madrid. All approved vaccines have demonstrated to be effective in reducing the risk of COVID, especially the risk of severe COVID-19 and hospitalization, leaving no doubts about the risk/benefit ratio of vaccination in the current pandemic (18). Our findings also support these recommendations. In our cohort, 88.4% of pwMS have been vaccinated, similar to the 86.7% vaccination rate of the general population in Madrid until 28 September 2021. In Spain, the vaccination has had good acceptance, with high vaccination rates since the beginning of the campaign.

In our cohort, no differences were found in demographic characteristics, MS clinical profile, MS activity, DMT, and basal lymphocyte count between COVID+ and COVID– subgroups. Zabalza et al. through an email survey with 758 valid respondents determined that age, contact with a confirmed case, residence in Barcelona, MS duration, and time on CD20 treatment were independent factors for presenting COVID-19 in a multivariable model (19). In our series, only smoking was associated with SARS-CoV-2 infection, as a protective factor. In the literature, data on the relationship between smoking and COVID-19 are contradictory and inconclusive (20). Smoking is well-established as having an adverse impact on lung health, and some authors have described that patients with a smoking history have a higher likelihood of developing more severe symptoms of COVID-19 and worse in-hospital outcomes than non-smokers (21). In contrast, the prevalence of current smokers among hospitalized patients with COVID-19 has been reported consistently lower than that observed in the general population (22, 23) and, consequently, some authors conclude that current smokers appear to be at a reduced risk of SARS-CoV-2 infection, compared with non-smokers (22–24). The association between tobacco and SARS-CoV-2 infection seems to be complex. On the one hand, tobacco may worsen the prognosis of those patients with a long history of smoking but may behave as a protective factor in patients with a short history of tobacco smoking who have not yet developed lung pathology. Although the mechanism involved in this protective factor is poorly understood, Polverino et al. postulated that cigarette smoke or nicotine stimulation may modify angiotensin-converting enzyme 2 (ACE2) expression (22). It cannot be ruled out a direct effect of tobacco smoke. The oxidizing effect of tobacco smoke or the heat emitted by the cigarette could also have a viricidal effect. In any case, this association may not imply a true or causal relationship, and smoking is not advocated as a prevention or treatment of COVID-19 (24).

In our cohort, EDSS ≥3.5 and pulmonary comorbidity were associated with more severe forms. In the North American Registry, increased disability (defined as non-ambulatory) was independently associated with hospitalization, as well as age, black race, cardiovascular disease, diabetes, and obesity (25). During the first wave, Loonstra et al. reported that among the Dutch population, pwMS with COVID-19 who were hospitalized were older, were more often male, and had secondary-progressive MS, higher EDSS score, and more comorbidity compared to nonhospitalized patients (13). They did not find any association between severity of COVID-19 and low lymphocyte count, as we did not find (13). In the French Covisep registry, Louapre et al. detected that age, EDSS ≥ 6, and obesity were independent variables for severe COVID-19 forms in the multivariate logistic regression model (14).

A total of 12 (13.9%) patients were hospitalized in our series, whereas Sahraian et al. reported 25% (2) and Parrotta reported 24% (3) of COVID hospitalization rate in pwMS. Our data are closer to the admission rate in the general population of Madrid, where 13.85% of infected patients required hospitalization as of 28 September 2021. Nevertheless, this data depend on the diagnostic capacity of each region and should be viewed with caution. Landtblom et al. also reported a similar risk of more severe COVID-19 outcomes in pwMS compared to the general population in the Swedish MS registry (SMSreg) (10).

In our cohort, one (1.2% of total and 1.7% of confirmed cases) patient died. This percentage is similar to 1.54% reported by Sormani et al. in the Italian Register (15) but lower than 2.3% (5/219) reported by Moreno-Torres et al. (26) and 3.5% observed in the French Covisep registry (14). The only death observed in our cohort took place during the second wave, whereas French Register published data only until 21 May 2020 and Italian Register published data only until 10 September 2020. Therefore, these results are not fully comparable. Our death rate is also lower than that observed in the general population of Madrid until 28 September 2021 (2.79% of infected patients died). These differences may be explained by the fact that the rates have not been stratified by age and sex. Higher COVID mortality rates have been observed in older population, while MS predominantly affects women aged between 20 and 40 years.

Our study has several limitations. Due to its observational nature, some data were missing and could not be analyzed. Another problem was the lack of access to testing in our area during the first coronavirus wave (as all over the world). The inclusion of all COVID-19 cases (possible, probable, and confirmed) in the study of risk factor associated with infection and with severe forms makes these results less reliable. Finally, as a consequence of its unicentric design, the sample size is limited. Larger studies with more extensive populations may better elucidate other COVID implications in the population with MS.

## Conclusion

This study provides several important observations. The cumulative incidence of confirmed COVID-19 in pwMS is similar to that of the general population, with a higher incidence during the first wave and a lower incidence during the second to fifth waves. Only smoking was associated with the risk of having COVID-19 as a protective factor. Other comorbidities, MS clinical profile, and DMT were not risk factors. The clinical outcome of pwMS with COVID-19 is good, with 86% mild forms. Higher EDSS and pulmonary comorbidities were associated with severe forms of COVID-19.

## Data availability statement

The raw data supporting the conclusions of this article will be made available by the authors, without undue reservation.

## Author contributions

BP and YA: conceptualization, data curation, formal analysis, methodology, visualization, writing—original draft preparation, and writing—review editing. JG: formal analysis, methodology, visualization, writing—original draft preparation, and writing—review editing. GM and AM: data curation and software. LR: conceptualization and data curation. IT: formal analysis and methodology. MT: data curation. All authors contributed to the article and approved the submitted version.

## Conflict of interest

The authors declare that the research was conducted in the absence of any commercial or financial relationships that could be construed as a potential conflict of interest.

## Publisher's note

All claims expressed in this article are solely those of the authors and do not necessarily represent those of their affiliated organizations, or those of the publisher, the editors and the reviewers. Any product that may be evaluated in this article, or claim that may be made by its manufacturer, is not guaranteed or endorsed by the publisher.
